# Research on shock wave driving technology of methane explosion

**DOI:** 10.1038/s41598-024-65797-5

**Published:** 2024-06-28

**Authors:** Chao-yuan Huang, Fei Liu, Kai Xin, Yong-hong Gao, Ya-peng Duan

**Affiliations:** 1 Institute of Defense Engineering, AMS, PLA, Luoyang, 471023 China; 2 Institute of Defense Engineering, AMS, PLA, Beijing, 100850 China

**Keywords:** Explosion wave simulation equipment, Detonation drive, Shock wave load, FLACS, Engineering, Civil engineering

## Abstract

In order to improve the driving ability of the explosion wave simulation equipment, reduce the erosion effect of condensed explosives on the explosion wave simulation equipment, improve the safety of the test process, and make better use of the meteorological detonation driving method, it is necessary to optimize the source of the shock wave load in the driving section. Based on the finite volume method of FLACS, a methane detonation driving model corresponding to the test is established to explore the feasibility of using methane as an explosion source to test the structure against explosion shock wave. A methane detonation drive test was carried out to verify the accuracy of the numerical model. Finally, an engineering model for attenuation of shock wave overpressure peak value in pipeline is established by dimensional analysis, and the model coefficient is determined by numerical simulation and test data. The results show that the blast pressure is the highest when the methane volume ratio reaches 9.5 vol% in the methane-air mixture. Simply increasing oxygen content has little effect on the peak overpressure and positive pressure duration of shock wave. In the pure oxygen environment, the detonation effect can be achieved when the volume ratio of methane to oxygen is 1:2, and the incident pressure of the shock wave is proportional to the volume of the gas cloud. When the gas cloud volume is constant, a reasonable selection of methane-oxygen mixture ratio can achieve a better detonation effect, which can effectively increase the peak overpressure of the shock wave in the test section. The research results can provide technical reference for the development of new explosion wave simulation equipment.

## Introduction

In the field of explosion shock wave simulation, achieving a more desirable effect can be accomplished by utilizing high-pressure gases^1–5^ and high-energy explosives such as TNT^6–8^ as the loading source for simulating explosion waves. However, there exists a bottleneck in the loading capacity of the equipment when dealing with shock waves that have high loads and long holding times. On one hand, as the initial pressure increases in the explosion chamber section, there is an exponential increase in compressor power requirements resulting in longer pressurization time and reduced efficiency. On the other hand, increasing the amount of TNT explosives within the explosion chamber leads to higher structural loads caused by air shock waves generated during explosive detonation, thereby compromising safety. Considering these two issues and aiming to meet design requirements for load sources in explosion wave simulation equipment, it becomes necessary to fulfill certain conditions: (1) storage and utilization with high stability; (2) generation of controllable loads; (3) causing less damage to explosion simulation equipment compared to high-energy explosive load sources. Based on these conditions, methane gas is investigated as a potential load source.

Aiming at the load changes generated by combustible gas explosion, relevant scholars at home and abroad have conducted a large number of studies. In the experimental aspect, Peng Pei et al.^9^ studied the deformation and damage mode of autoclaved aerated concrete masonry wall under the action of gas explosion. The effect of gas explosion loading on the height and thickness of the masonry wall was analyzed and a reinforcement scheme was given. Yang et al.^10^ conducted comparative experiments on methane-air premixed gas with a methane volume fraction of 9.5 vol % under different working conditions. The results proved that the pipe outlet blockage ratio was positively correlated with the peak explosion overpressure and explosion index. Wang et al.^11^ investigated the methane flammability limit, the maximum explosion pressure propagation and the explosion wave energy distribution, and the results proved that there was a volumetric effect on the flammability limit of methane, which was 9 vol %–17 vol % in a slender pipe with a volume of 76 dm^3^. The deflagration mode of methane within the flammability limit can be categorized into strong deflagration mode and weak deflagration mode. On this basis, Wu Jiansong et al.^12^ studied the comprehensive pipeline corridor gas explosion experimental system to study the effect of methane explosion overpressure distribution. The results table proved that the methane volume fraction of 9.5 vol% when the explosion overpressure peak is the highest. For the effect of other material components on methane combustion and explosion, Wang et al.^13^ conducted a hydrogen-methane-air mixture of pipeline deflagration experiments. The results proved that the first overpressure peak in the ventilated chamber was more than not the largest, and the overpressure peak first increased and then decreased. Yuan et al.^14^ investigated the inhibition effect of TFEP on the explosion of methane-air premixed gases with a concentration of 9.5 vol %, and explored the optimal filling conditions of TFEP. `lvarez-FernA`ndeza et al.^15^ investigated the explosion of methane-air premixed gases with different explosion chamber volumes in the explosive loading of methane-air mixtures was analyzed for the maximum peak pressure that could be reached and the velocity of the shock wave. From this, it was possible to determine whether deflagration or explosion had occurred. Wang Dai et al.^16^ determined the integrated pipeline corridor load distribution through the field corridor gas explosion test, the results proved that the measured peak overpressure of the field corridor gas explosion test is as high as 0.63 MPa, and the duration of the ramp-up and ramp-down are about 0.1 s. Yue et al.^17^ carried out a methane explosion test in a full-size residential building of 105 m. The results proved that the peak overpressure of the gas explosion of the residential building is at around 10 kPa. For the study of explosive power of gaseous gases. Subburaj et al.^18^ used diaphragm-free surge tubes to study the explosive power of methane and n-hexane, which opens up new possibilities for reliable chemical kinetic studies.

In terms of numerical simulation, Zhu et al.^19^ investigated the effect of shock wave oscillations on the peak overpressure generated by the explosion of a methane-air mixture pair using AutoReaGas 3D software. The results proved that the oscillation period increased with the increase of pipe length. Zhang Xiuhua et al.^20,21^ explored the possibility of explosive shock loading of combustible gases as an explosive source by using LS-DYNA software. Zhang et al.^22^ investigated the explosion of different proportions of methane-hydrogen mixtures inside tunnels by using the gas explosion simulation software FLACS. The results proved that with the increase of the proportion of H2, the same volume of the gas cloud will produce a higher peak overpressure. Cao et al.^23^ used numerical simulation to study the effect of methane concentration on the internal pressure of the pipeline. When the concentration was 7 vol%, the pressure time-course curve showed almost no significant oscillations. However, when the concentration of 9-13vol %, the pressure time-course curve of the oscillation is more obvious. Chen et al.^24^ studied the methane-air mixture explosion in a long straight space, and established the relevant numerical model. The results proved that in the 20m long straight space with obstacles in the gas explosion, with the increase of the roof pressure in the space, the shock wave overpressure peak also increases. Cao et al.^25^ simulated the methane-air mixture in the concentration of a certain time, the leakage of the explosion process, and the low concentration of gas in the pipeline explosion peak overpressure was observed and compared. Summarized the explosion characteristics of low concentration of gas in the pipeline transportation process.

In summary, the majority of existing research focuses on studying gas explosions as destructive loads under natural conditions, with a primary emphasis on reducing personnel injuries and structural damage. However, there is limited research on increasing the output load of gas a given volume. The findings of this study can serve as a technical reference for simulating explosion wave tests using gas as a source of load.

## Numerical calculation model

Driving device within the gas-air mixture from the bottom of the ignition triggered by combustion and explosion, to the process of detonation of methane-air and methane-oxygen mixtures in the rapid reaction of the flame to move forward, the value of the air pressure is increasing, need to undergo a complex evolutionary process. This process is coupled with the underlying chemical reaction, flame, temperature, turbulence development, pressure wave propagation, flame and pressure wave interactions and other complex processes, involving chemistry, thermodynamics, wave dynamics, heat and mass transfer and fluid dynamics and many other disciplines, it is a non-stationary non-stationary and occur in tens or even hundreds of milliseconds of time in the rapid explosion process.

This paper uses FLACS software based on the finite volume method to establish a numerical analysis model of gas explosion within the drive unit. Under the premise of extensive comparison with the test data and verification of its validity, it carries out the analysis of the propagation law of the shock wave within the driving device and the test section, and discusses the influence of methane concentration, volume and other factors on the relevant parameters of the gas explosion shock wave.

### Mechanism of methane explosion reaction

Methane concentration, methane combustion and explosion process is a very complex chemical reaction process, for methane combustion, detonation and detonation of the three stages, usually using the chain reaction theory to analyze. Chain reaction theory believes that the methane explosion is due to the related reactant molecules are affected by external heat energy, decompose into several free radicals, and have a large activation energy, and then the chain reaction throughout the entire chemical reaction process. Free radicals gradually increase with the chain reaction, the chemical reaction speed is also gradually accelerated, when the accumulation of a certain amount, methane explosion. Among them, the chain reaction of methane explosion mainly consists of four parts: (1) initiating the chain reaction. (2) Transfer chain reaction. (3) Chain reaction dispersion. (4) The chain reaction terminates. The chain reaction mechanism of "methane-air" mixture at normal temperature and pressure is shown in Table 1.

**Table 1:** Reaction of methyl alkyl elements at normal temperature and pressure.

Serial number	Chemical reaction equation	Reaction stage
1	$CH_{4} + O_{2} \to \dot{C}O_{2} + HO_{2}$	Initiating reaction
2	$\dot{C}H_{3} + O_{2} \to CH_{2} O + \dot{O}H$	Transfer reaction
3	$CH_{4} + \dot{O}H \to \dot{C}H_{3} + H_{2} 0$	
4	$CH_{2} O + \dot{O}H \to H_{2} O + H\dot{C}O$	
5	$CH_{2} + O_{2} \to H\dot{O}_{2} + H\dot{C}O$	Dispersion reaction
6	$H\dot{C}O + O_{2} \to CO + H\dot{O}_{2}$	Transfer reaction
7	$H\dot{O}_{2} + CH_{4} \to H_{2} O_{2} + \dot{C}H_{3}$	
8	$\dot{H}O_{2} + CH_{2} O = H_{2} O_{2} + H\dot{C}O$	
9	$CH_{2} O \Leftrightarrow tubewall$	Termination reaction

### Governing equation

In FLACS software, a system of partial differential equations constructed from the mass and momentum conservation equations as well as the transport equations for enthalpy, fuel mass components, and mixed components are used to solve the fluid behavior of an ideal compressible gas flow. The mass conservation equation is:

1 $$ \frac{\partial }{\partial t}\left( {\beta_{\nu } \rho } \right) + \frac{\partial }{{\partial x_{j} }}\left( {\beta_{\nu } \rho u_{j} } \right) = \frac{{\dot{m}}}{v}, $$

The momentum conservation equation is:

2 $$ \frac{\partial }{\partial t}\left( {\beta_{\nu } \rho u_{i} } \right) + \frac{\partial }{{\partial x_{j} }}\left( {\beta_{\nu } \rho u_{i} u_{j} } \right) = - \beta_{\nu } \frac{\partial p}{{\partial x_{i} }} + \frac{\partial }{{\partial x_{j} }}\left( {\beta_{j} \sigma_{ij} } \right) + F_{o,i} + F_{w,i} + \beta_{\nu } \left( {\rho - \rho_{0} } \right)g_{i} , $$

3 $$ F_{o,i} = - \rho \left| {\frac{\partial \beta }{{\partial x_{i} }}} \right|u_{i} \left| {u_{i} } \right|. $$

The transport equation for enthalpy is:

4 $$ \frac{\partial }{\partial t}\left( {\beta_{\nu } \rho h} \right) + \frac{\partial }{{\partial x_{j} }}\left( {\beta_{j} \rho u_{j} h} \right) = \frac{\partial }{{\partial x_{j} }}\left( {\beta_{j} \frac{{\mu_{eff} }}{{\sigma_{h} }}\frac{\partial h}{{\partial x_{j} }}} \right) + \beta_{v} \frac{{D_{p} }}{{D_{t} }} + \frac{{\dot{Q}}}{V}. $$

The transportation equation for the fuel mass fraction is:

5 $$ \frac{\partial }{\partial t}\left( {\beta_{\nu } \rho Y_{fuel} } \right) + \frac{\partial }{{\partial x_{j} }}\left( {\beta_{j} \rho u_{j} Y_{fuel} } \right) = \frac{\partial }{{\partial x_{j} }}\left( {\beta_{j} \frac{{\mu_{eff} }}{{\sigma_{fuel} }}\frac{{\partial Y_{fuel} }}{{\partial x_{j} }}} \right) + R_{fuel} . $$

The transport equation for the mixed components is:

6 $$ \frac{\partial }{\partial t}\left( {\beta_{\nu } \rho \xi } \right) + \frac{\partial }{{\partial x_{j} }}\left( {\beta_{j} \rho u_{j} \xi } \right) = \frac{\partial }{{\partial x_{j} }}\left( {\beta_{j} \frac{{\mu_{eff} }}{{\sigma_{\xi } }}\frac{\partial \xi }{{\partial x_{j} }}} \right). $$

The standard *k*-*ε* eddy viscosity model is used to describe turbulent disturbances. Where the transport equation for the turbulent kinetic energy (*k*) is:

7 $$ \frac{\partial }{\partial t}\left( {\beta_{\nu } \rho k} \right) + \frac{\partial }{{\partial x_{j} }}\left( {\beta_{j} \rho u_{j} k} \right) = \frac{\partial }{{\partial x_{j} }}\left( {\beta_{j} \frac{{\mu_{eff} }}{{\sigma_{k} }}\frac{\partial k}{{\partial x_{j} }}} \right) + \beta_{\nu } P_{k} - \beta_{\nu } \rho \varepsilon . $$

8 $$ \mu_{eff} = \mu + \rho C_{\mu } \frac{{k^{2} }}{\varepsilon }. $$

The transport equation for the turbulent kinetic energy dissipation rate (*ε*) is:

9 $$ \frac{\partial }{\partial t}\left( {\beta_{\nu } \rho \varepsilon } \right) + \frac{\partial }{{\partial x_{j} }}\left( {\beta_{j} \rho u_{j} \varepsilon } \right) = \frac{\partial }{{\partial x_{j} }}\left( {\beta_{j} \frac{{\mu_{eff} }}{{\sigma_{\varepsilon } }}\frac{{\partial_{\varepsilon } }}{{\partial x_{j} }}} \right) + \beta_{v} P_{\varepsilon } - C_{2\varepsilon } \beta_{v} \rho \frac{{\varepsilon^{2} }}{k}, $$

10 $$ P_{\varepsilon } = C_{1\varepsilon } \frac{\varepsilon }{k}P_{k} \left( {1 + C_{3\varepsilon } R_{f} } \right), $$

11 $$ R_{f} = - \frac{{G_{b} }}{{P_{k} }}\frac{{\left| {\overrightarrow {u} \times \overrightarrow {g} } \right|}}{{\left| {\overrightarrow {u} } \right|\left| {\overrightarrow {g} } \right|}}g. $$

In the above equations, *j* is the spatial index; *i* is the spatial index of a component; *β*_*v*_ is the volumetric porosity; *ρ* is the gas density; *ρ*_0_ is the initial gas density; *x* is the gas concentration; *β* is the conversion factor in the *β*-model; *m* is the mass ratio; *p* is the absolute pressure; *σ*_*ij*_ is the stress tensor; *u* is the velocity; *V* is the volume; *F*_*wi*_ is the resistance to flow at the wall;* F*_*oi*_ is the flow resistance due to the blocking of subgrid obstacles flow resistance; *ɡ* is the gravitational acceleration; h is the specific enthalpy; *σ* is the Prandtl-Schmidt number; *Q* is the heat flow rate; *Y*_*fuel*_ is the mass fraction of the fuel; *R*_*fuel*_ is the reaction rate of the fuel; *ξ* is the mixing component; *μ* is the kinetic viscosity; *C*_*μ*_ is a constant, taking the value of 0.09; *μ*_*eff*_ is the effective viscosity; *P*_*k*_ consists of the flow shear stress (*G*_*s*_), wall shear stress (*G*_*w*_), buoyancy (*G*_*b*_), and turbulent kinetic energy generation rate of subgrid obstacles (*G*_*o*_) together. Where *C*_1*s*_, *C*_2*s*_, and *C*_3*s*_ are constants taking values of 1.44, 1.92, and 0.8, respectively; *P*_*ξ*_ is the rate of generation of dissipation; and *R*_*f*_ is the buoyancy term.

### Geometric model and parameter setting

In the numerical simulation of the gas explosion drive, the following reasonable simplifications and assumptions are used: (1) each component of the gas is an ideal compressible flow; (2) the wall is smooth without deformation and does not take into account the heat transfer, ignoring the main vessel as well as the effect of the wall thickness of the test section on the results of the simulation; (3) the flow field inside the main vessel is stationary in the initial stage.

Some of the parameters were set identically in the model validation and the subsequent models developed. Among them, the initial temperature and initial ambient pressure were set to 20 °C and 100,000 Pa. A cubic Cartesian grid of the same size was used in each region of the simulation. In order to solve the momentum and continuity equations at the basin boundaries and to reduce the reflection behavior of the pressure wave at the basin boundaries, "PLANE WAVE" boundary conditions are used at each basin boundary in the semi-confined case. In order to choose the appropriate discrete time steps in the early stage of the explosion when the flow rate and combustion rate are low, and in the late stage of the explosion when the combustion rate accelerates rapidly, the Courant-Friedrich-Levy number CFLC based on the speed of sound and the CFLV based on the flow rate of the fluid are set to 5 and 0.5, respectively.

**Figure 1:**
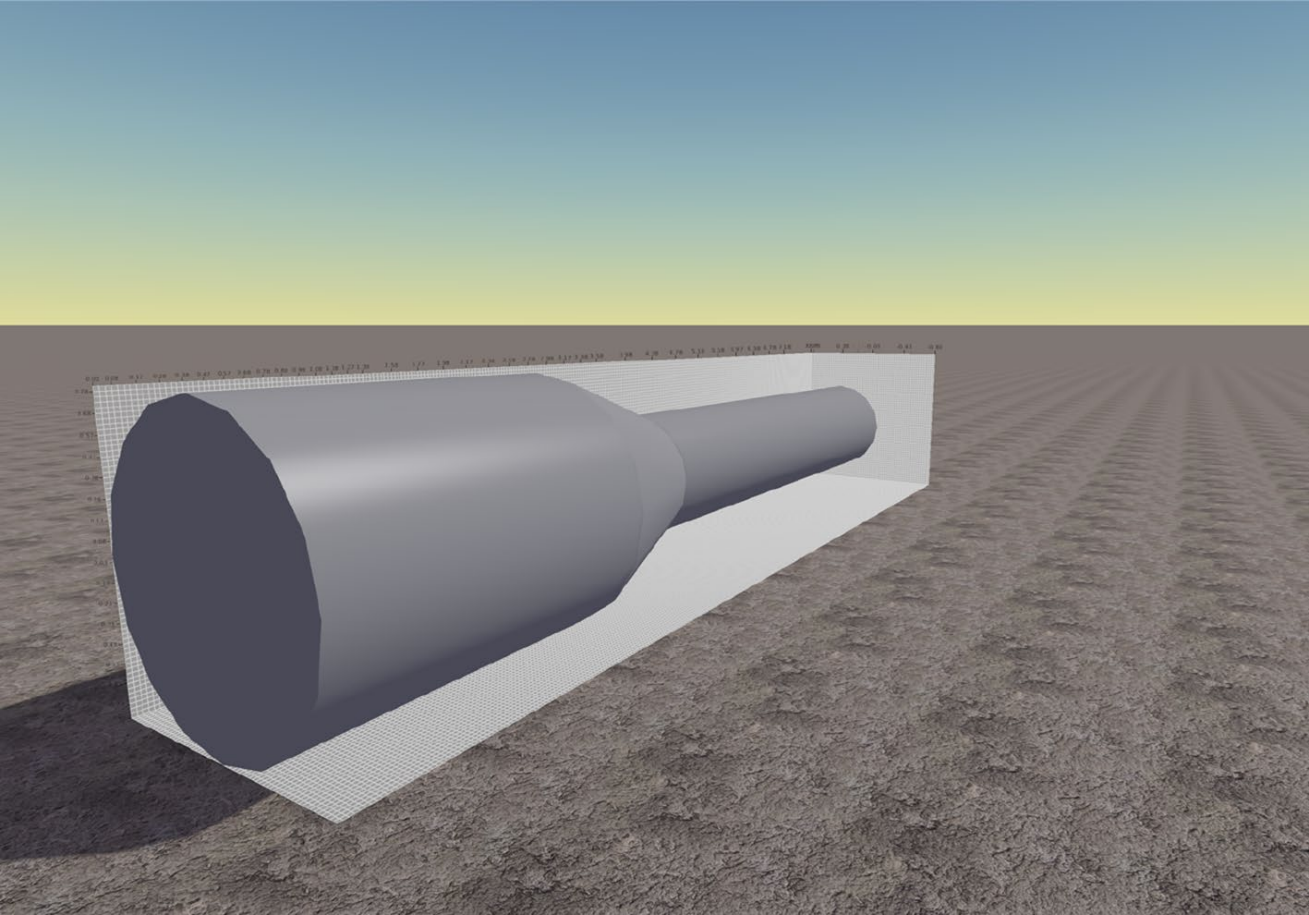
Numerical model of gas explosion driving.

In the modeling process, the methane volume change was achieved by setting up a rectangular gas cloud with the same cross-sectional dimensions but different lengths. To ensure the closure of the model, the wall thickness of the pipe is set to 0.2 m. In the semi-closed case, the size of the whole computational basin is 2 m (X-direction) × 2 m (Z-direction) × 8 m (Y-direction). The numerical calculation model is shown in Fig. 1.

### Grid convergence analysis

Two cases with different methane equivalents were selected for grid convergence tests to ensure stable and reliable results. The dimensions of the fuel area in the main vessel (2 × 0.5 × 0.5) were kept consistent during the calculations. A 1 m stretch at the exit of the test section (+ Y direction) was used to prevent large mass residuals from making the calculations non-convergent. Without the stretching region, uniform grid sizes were used for the grid convergence tests with grid sizes of 0.025m, 0.05m, 0.075, and 0.1m, and grid counts of 1,310,720, 163,840, 51,788, and 20,480, respectively.

**Figure 2:**
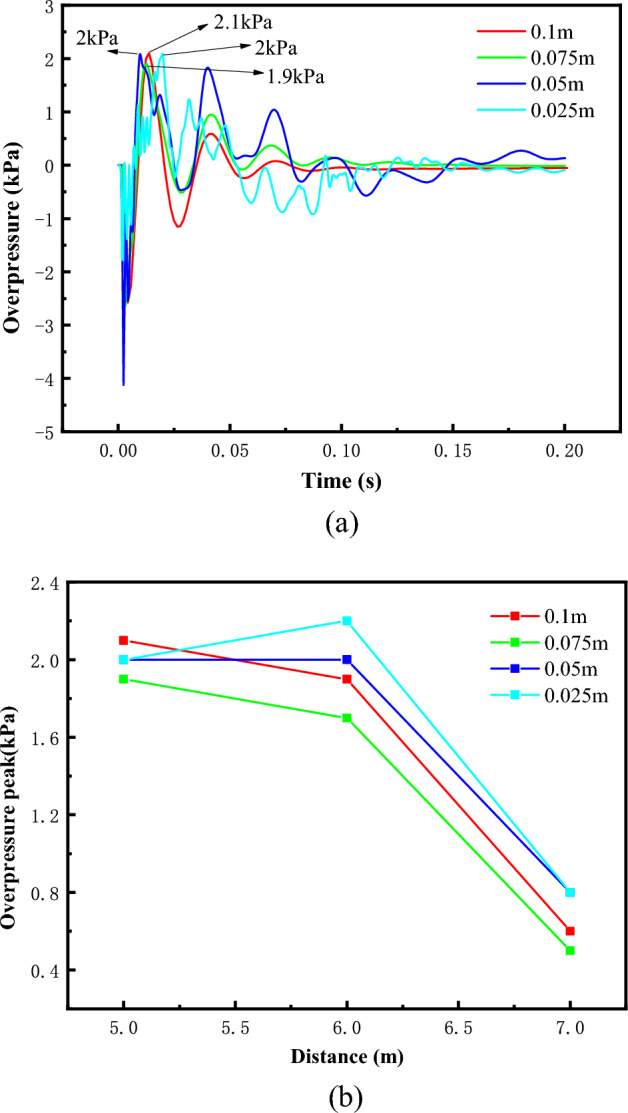
Methane-air mixture overpressure time course curve and overpressure peak change process. (**a**) Time course of overpressure at T1 measurement point, (**b**) Variation of peak overpressure with distance at different measurement points.

Figure 2a shows the overpressure time-course curves for methane-air mixture at methane concentration of 9.5 vol % at T1 measurement point with different grid sizes, and (b) shows the variation of peak overpressure with distance. The peak overpressure values measured for grid sizes 0.025 m and 0.05 m are equal, with errors of 5.26% and 5.0%, respectively, compared to grid sizes 0.075 m and 0.1 m. The overpressure peak values measured for grid sizes 0.025 m and 0.05 m are equal, with errors of 5.26% and 5.0%, respectively. However, the curve ramping up process is slow and the pressure oscillations are too frequent when the grid size is 0.025m. Comparison of the relationship between the peak overpressure with distance found that with the increase of distance, the coarser the grid size the smaller the peak overpressure, the maximum error of the peak occurred at 6m, 29.4%. The reason for the decrease of the peak overpressure with the increase of the grid size is that it is difficult to accurately deal with the change of pressure gradient in the test section with a too coarse grid.

**Figure 3:**
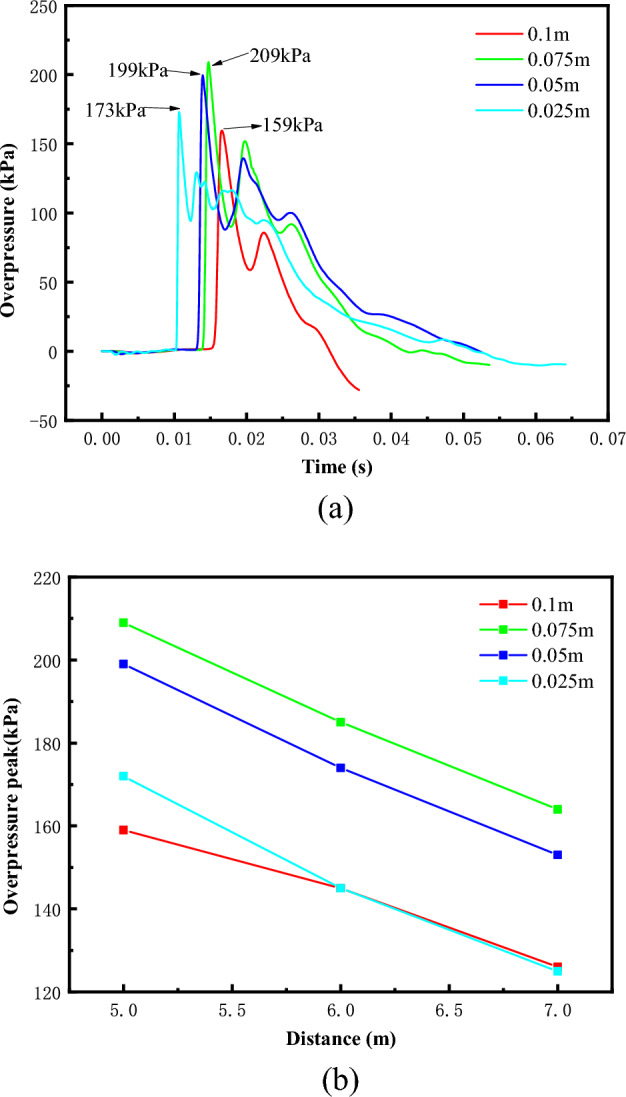
Overpressure time history curve of methane-oxygen mixture and overpressure peak change process. (**a**) overpressure time history curve of T1 measuring point, (**b**) Peak overpressure at different measuring points.

Figure 3a shows the overpressure time course curves of methane-oxygen mixtures with different grid sizes for the T1 measurement point at a methane concentration of 33.3 vol %, and (b) shows the variation of the peak overpressure with distance. A grid size of 0.025 m minimizes the arrival time of the shock wave, but its peak overpressure is lower. Grid sizes of 0.05m and 0.075m have closer shock wave arrival times as well as peak overpressure values. According to the relationship between the peak overpressure value and the distance, it can be seen that the peak overpressure value at each measurement point is the largest when the grid size is 0.075m, and the average errors between the grid sizes of 0.1, 0.05, and 0.025 and the maximum peak values are 22.8%, 5.7%, and 21.0%, respectively. In summary, the selection of grid size 0.05m is reasonable in terms of calculation accuracy as well as efficiency.

### Influence factors of shock wave overpressure peak

#### Methane concentration

**Figure 4:**
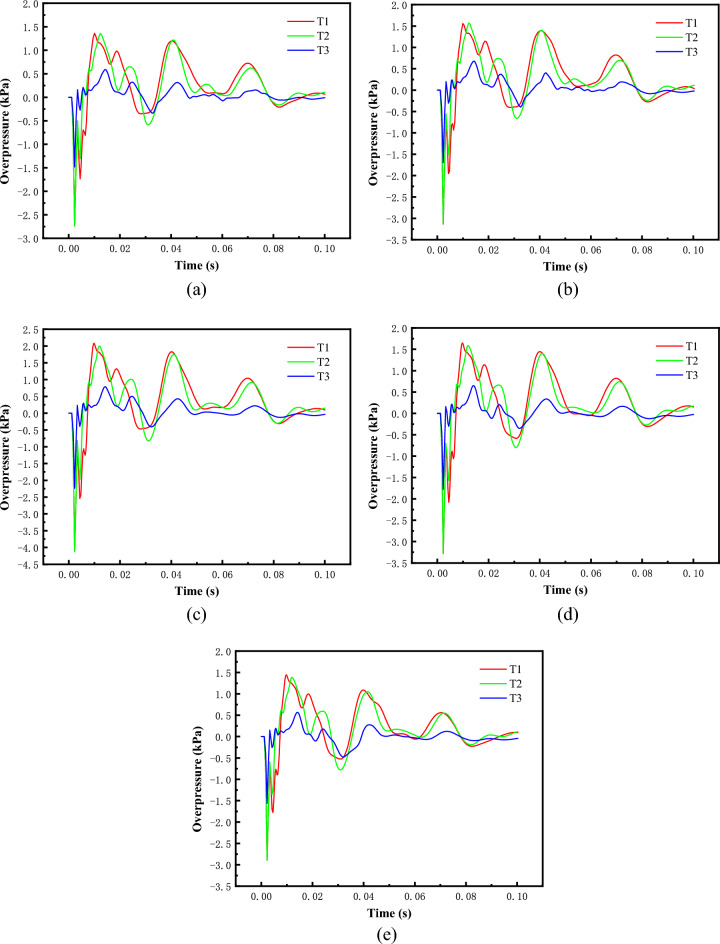
Overpressure time history curve of shock wave with different methane concentrations. (**a**) Methane concentration 7.5vol %, (**b**) Methane concentration 8.5vol %, (**c**) Methane concentration 9.5vol %, (**d**) Methane concentration 10.5vol %.

In air, different methane concentration explosion generated pressure is different. In order to explore in the main pressure vessel, different concentrations of methane—air mixture explosion, in the test section of the peak overpressure generated change rule. Methane concentration of 7.5 vol %, 8.5 vol %, 9.5 vol %, 10.5 vol % and 11.5 vol %, five conditions for numerical calculations. In the calculation process, make the methane—air mixture volume certain (0.5m3), in the test section set up three measurement points, respectively, from the bottom of the drive equipment 5m, 6m and 7m, Fig. 4 for different concentrations of methane—air mixture explosion numerical simulation results.

Table 2 shows the comparison of the peak value of shock wave overpressure under different working conditions. The results show that with the increase of methane consumption, the peak value of incident shock wave overpressure acting on the test section of the driving equipment increases nonlinearly, but the increase of positive pressure acting time is not obvious. Under the same working condition, the first peak value of incident overpressure is the highest, and the attenuation speed of the subsequent peak value is gradually accelerated. The maximum error of the peak value of shock wave overpressure at the same measuring point with different methane concentrations is 34.6%, 32.3 and 29.1%. When the concentration of methane-air mixture is 9.5vol %, the peak of shock wave incident overpressure reaches the maximum, which is basically consistent with the conclusions obtained in literatures Refs.^12^ and^14^.

**Table 2:** Peak overpressure of shock wave with different methane concentrations in methane-air mixture.

Test condition	Air usage (m^3^)	Methane consumption (m^3^)	Methane concentration (vol%)	T1	T2	T3
				*P*_1_ (kPa)	*P*_2_ (kPa)	*P*_3_ (kPa)
1	0.4625	0.0375	7.5	1.36	1.34	0.58
2	0.4575	0.0425	8.5	1.57	1.56	0.67
3	0.4525	0.0475	9.5	2.08	1.98	0.79
4	0.4475	0.0525	10.5	1.64	1.59	0.65
5	0.4425	0.0575	11.5	1.44	1.38	0.56

(**e**) Methane concentration 11.5vol %.

#### Different oxygen content

**Figure 5:**
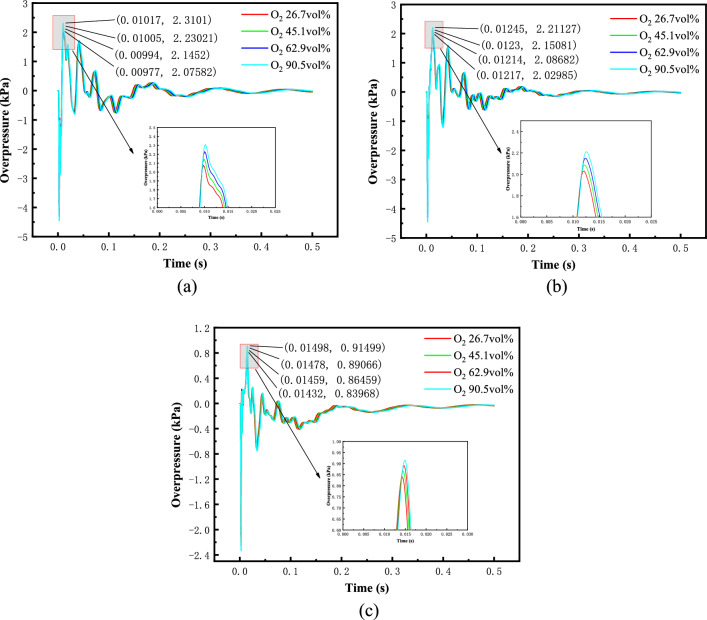
Overpressure time history curve of each measuring point under different working conditions. (**a**) T1, (**b**) T2, (**c**)T3.

Oxygen as a methane explosion reactant, different oxygen content on the methane explosion generated by the shock wave overpressure has a greater impact. Selected in the gas cloud volume (0.5 m^3^) and methane concentration (9.5 vol %) a certain amount of different oxygen content on the methane explosion generated by the peak shock wave overpressure simulation. Figure 5 shows the oxygen content in 26.7 vol %, 45.1 vol %, 62.9 vol % and 90.5 vol %, the time course of the shock wave overpressure curve at each measurement point. As can be seen from the figure, due to the shock wave propagation velocity of less than 340 m/s, or is a deflagration phenomenon, and did not meet the requirements of the explosive blast. When the oxygen content increases, compared with in the air, the shock wave overpressure rise significantly faster, but the positive pressure duration rise is not obvious. With the increase in oxygen content, the shock wave overpressure peak gradually increased, but the decay rate is basically the same as in the air.

#### Gas cloud volume effect

**Figure 6:**
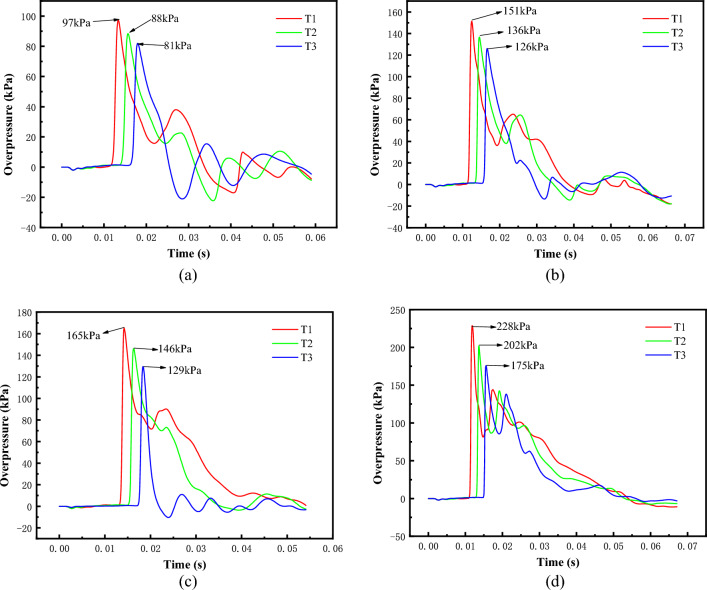
Overpressure time history curve of shock wave with different gas cloud volume. (**a**) 0.125m^3^, (**b**) 0.25m^3^, (**c**) 0.375m^3^, (**d**) 0.5m^3^.

**Figure 7:**
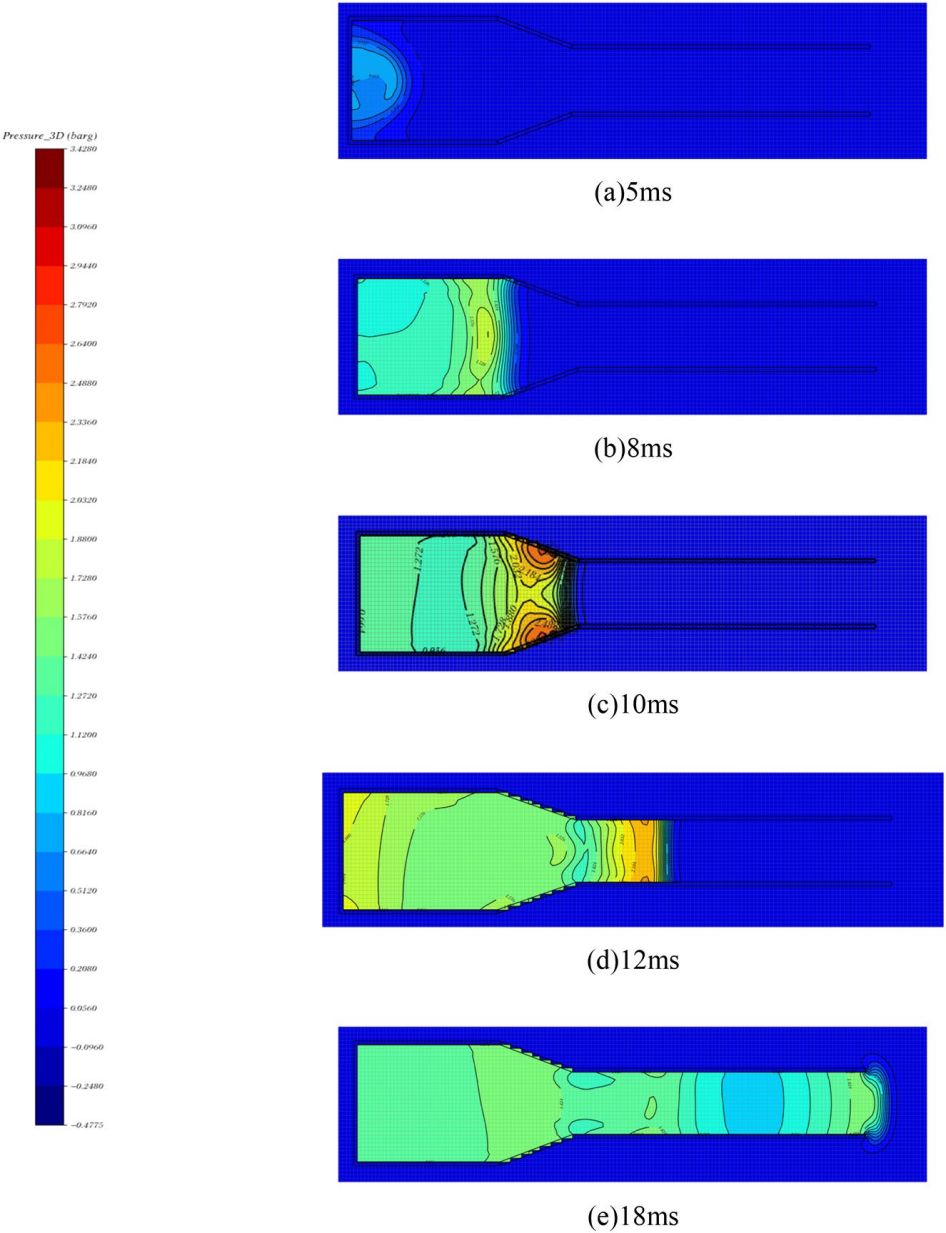
Pressure cloud image during shock wave propagation. (**a**) 5ms, (**b**) 8ms, (**c**)10 ms, (**d**)12 ms, (**e**)18 ms.

The methane content as well as the oxygen content are the key factors in generating high loads from methane detonation. In order to explore the maximum power of methane bombardment under a certain equivalent, according to the complete reaction equation of methane CH_4_ + 2O_2_ = CO_2_ + 2H_2_O, it is determined that the volume ratio of methane-oxygen mixtures is 1:2 under the environment of pure oxygen, and the volumes are 0.125 m3, 0.25 m3, 0.375 m3 and 0.5 m3 for numerical simulation. Figure 6 shows the overpressure time course curves of each measurement point under different volumes. From the figure, it can be seen that the shock wave propagation speed is 558 m/s, which is greater than the sound speed of 340 m/s, and reaches the effect of bursting. The gas medium in the test section experienced three stages of pressurization, depressurization and aftershock oscillation. Table 3 for the methane-oxygen mixture of different gas cloud volume shock wave overpressure peak. With the increase of shock wave propagation distance, the peak shock wave overpressure and positive pressure duration show a decreasing trend. The larger the gas cloud volume is, the larger the peak overpressure is at each measurement point, and the longer the duration of positive pressure is. The attenuation coefficient of the shock wave peak overpressure in the unit length is maintained at about 0.9. Figure 7 shows the pressure cloud of the shock wave propagation process, from which it can be seen that when the shock wave reaches the test section, it basically forms a plane wave, which can be used for the loading test of the explosion wave simulation equipment.

**Table 3:** Peak shock wave overpressure of different gas cloud volumes in methane-oxygen mixture.

Gas cloud volume (m^3^)	Oxygen dosage (m^3^)	Methane consumption (m^3^)	Methane concentration (vol%)	T1	T2	T3
				*P*_1_ (kPa)	*P*_2_ (kPa)	*P*_3_ (kPa)
0.125	0.0833	0.0417	33.3	97	88	81
0.25	0.1666	0.0834	33.3	151	136	126
0.375	0.2499	0.1251	33.3	165	146	129
0.5	0.3332	0.1668	33.3	228	202	175

## Test verification

### Test system

**Figure 8:**
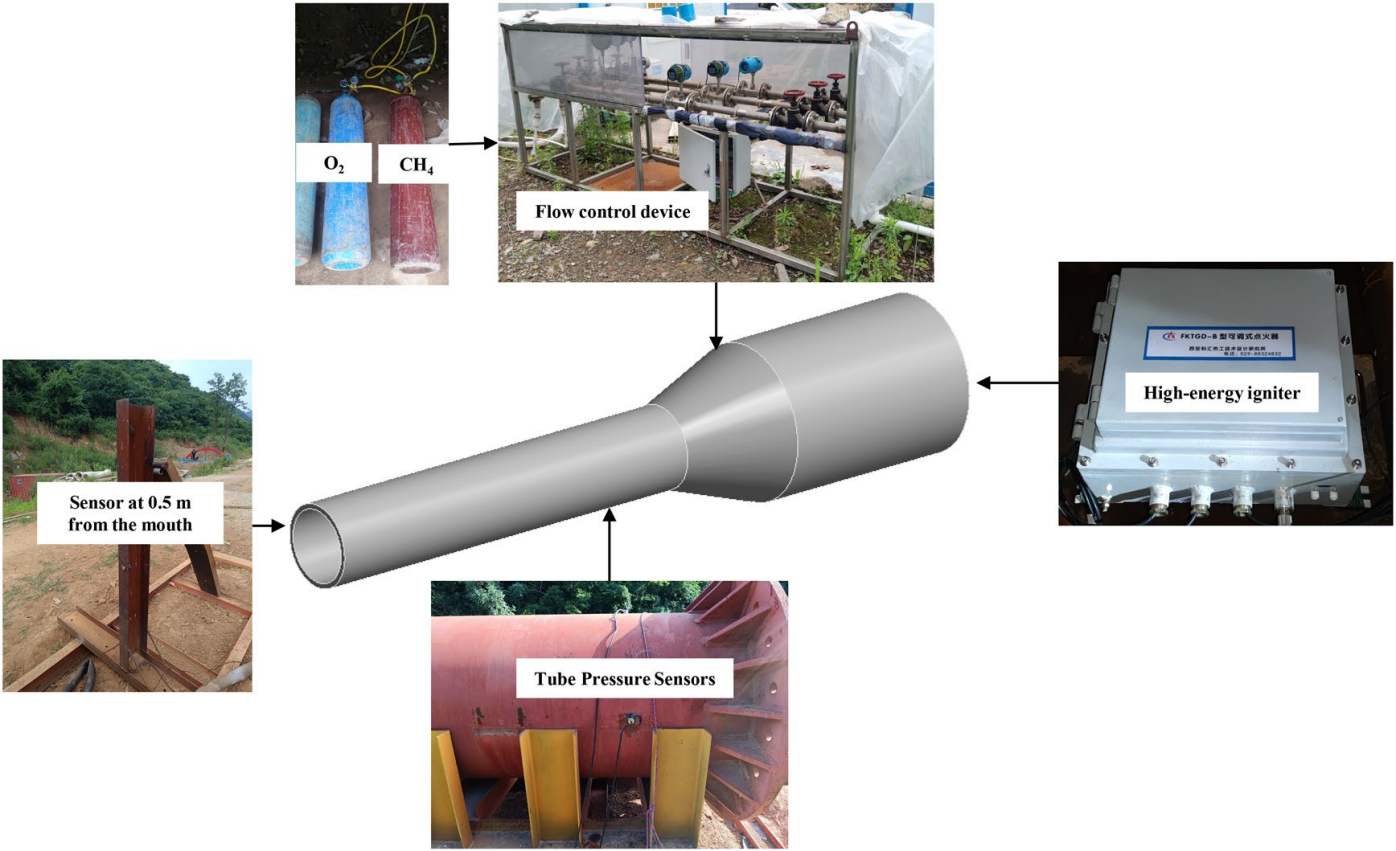
Test system.

The gas explosion drive test system consists of drive equipment, high-energy igniter, methane-oxygen cylinder set, sensors, data collector and drone together, as shown in Fig. 8. The pressure vessel structure is all steel, connected by welding and flange bolts. The main structure includes five main parts: the main pressure vessel, 1# test section, 2# test section, pressure vessel fixed bracket, and test section track. The main pressure vessel is made of Q345R steel plate and consists of a cylindrical steel drum with a length of 2000 mm and a diameter of 1500 mm and a conical steel drum with a length of 1000 mm and a diameter of 1500 mm and a variation of 800mm. 1# and 2# test sections are also made of Q345R steel plate, with a length of 2000 mm, an inner diameter of 800 mm and a thickness of 20 mm. Add 3 mm thick rubber ring to ensure the sealing of the whole vessel. The outer surface of the test equipment is coated with lacquer to enhance its corrosion resistance.

**Figure 9:**
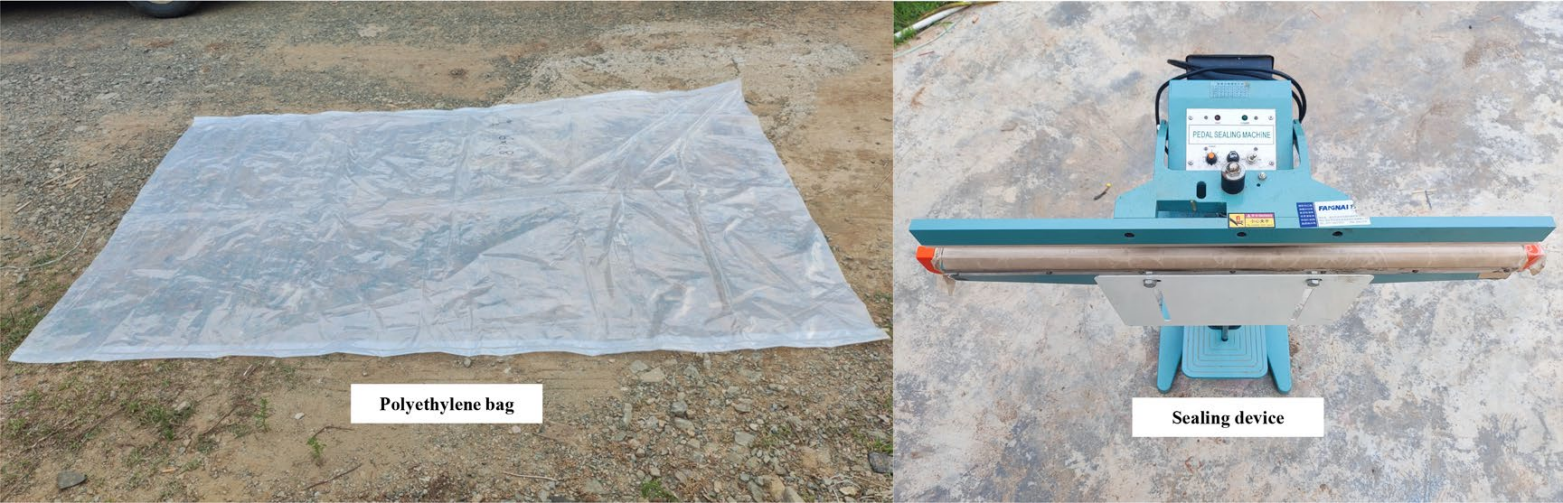
Inflatable bag and sealing device.

In this test, methane was used as the combustible gas and oxygen as the combustion gas. The 0.5 m^3^ gas bag in the test vessel was inflated through the bottle set, and at the same time the disposable explosion-proof fan was turned on to obtain a methane-oxygen mixture with uniform concentration. Before the test, the 0.5 m^3^ polyethylene bag was calibrated to ensure that a methane-oxygen concentration consistent with the test design was obtained (methane 33.3 vol %, oxygen 66.7 vol %). The ignition was carried out using an FKTGD-B adjustable high-energy igniter, with the ignition position 500 mm from the bottom of the pipe, using a remote ignition device to excite the 21J ignition head to realize the ignition. The inflatable bag and sealing device are shown in Fig. 9.

**Figure 10:**
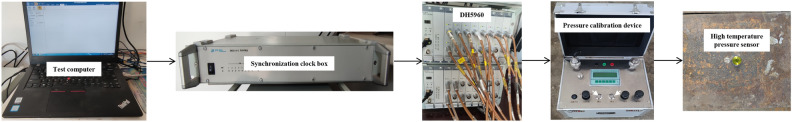
Test system.

**Figure 11:**
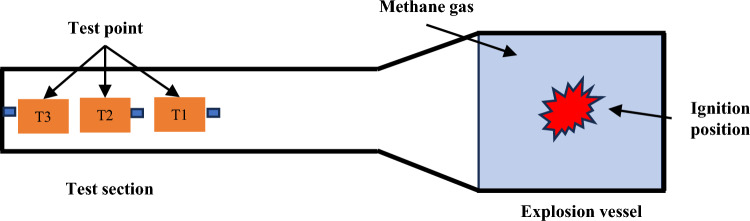
Layout of test pressure measuring points.

DH5960 ultra-dynamic signal test and analysis system is used for test data acquisition, which has a built-in hard disk, a transient sampling frequency of up to 10MHz, external trigger, signal trigger and other functions, and the pressure signal of each test point is transmitted through a low-noise signal cable. The test system is shown in Fig. 10. The test sampling frequency of 500 kHz. 3 range of 2Mpa piezoresistive high-frequency pressure sensor (DH4100) installed in the test section of the inner wall, measuring the explosion generated by the overpressure time curve, respectively, from the bottom of the drive section 5 m, 6 m, 7 m (T1–T3). The arrangement of measurement points is shown in Fig. 11.

### Test procedures and safety specifications

The test process can be specifically summarized as follows:

1. Install, adjust and confirm each part of the sensor, and check the installation and status of the trigger wire and the detonation wire.
2. Tie the high energy ignition head to 0.5 m^3^ polyethylene bag (2 0.5 0.5) 0.5 m from the bottom, lay the bag flat in the main pressure vessel, and align the bottom.
3. Fill the bag with 0.165 m^3^ oxygen first, then 0.165 m^3^ methane gas, and finally 0.17 m^3^ oxygen, and open the disposable explosion-proof fan to make the gas in the container evenly mixed.
4. The data acquisition instrument was balanced and zeroed out. At the same time, the remote control voltage of the high energy igniter was adjusted to 200 V, and the fan was shut down to keep the gas in the container stationary for 5 min.
5. A portable mobile power source is used to send out the initiation signal to the high energy igniter and record the overpressure time history curve during the explosion.

The test safety specifications are as follows:

1. Experimental site: methane explosion test is carried out in the designated field test site. The experimental site has good ventilation conditions and appropriate safety facilities to prevent injury and damage in the event of an explosion.
2. Personal protection: Personnel conducting methane explosion tests should wear appropriate personal protective equipment, such as safety glasses, protective gloves, protective clothing, etc.
3. Equipment safety: The equipment for the methane explosion test meets the relevant safety technical regulations and standards, and should be inspected and maintained regularly. Clear warning signs should be marked on the test equipment to remind people to pay attention to safety.
4. Explosion process control: 30 min before the explosion test, all test personnel evacuate to a safe area, check the measurement system again, and the detonation operator check the detonation line again.
5. Start to inflate, test staff to detect the inflation pressure, if abnormal, stop the inflation, if necessary to release the methane gas in the drive section.
6. Emergency treatment of explosion accidents: Formulate corresponding emergency treatment measures when conducting methane explosion tests. The experimental site is equipped with appropriate fire extinguishing equipment and first aid equipment to deal with possible explosion accidents.
7. After the test is completed, ventilate the inside of the drive section and clean the test site.

### Test results and analysis

#### Measured shock wave curve

**Figure 12:**
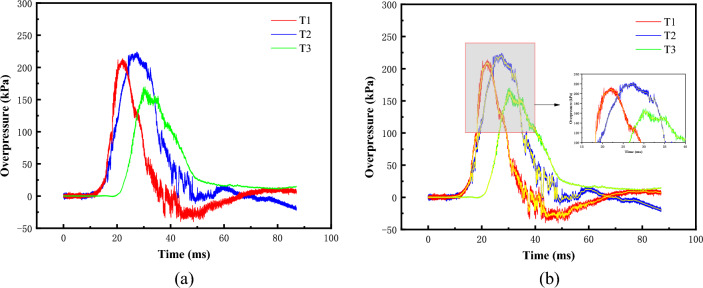
Test data. (**a**) Raw acquisition data, (**b**) 200Hz low-pass FFT filter.

Before analyzing the experimental results, the original data were low-pass filtered at 200 Hz to achieve smooth denoising of the original curves, as shown in Fig. 12.

#### Development characteristics of gas explosion load

**Figure 13:**
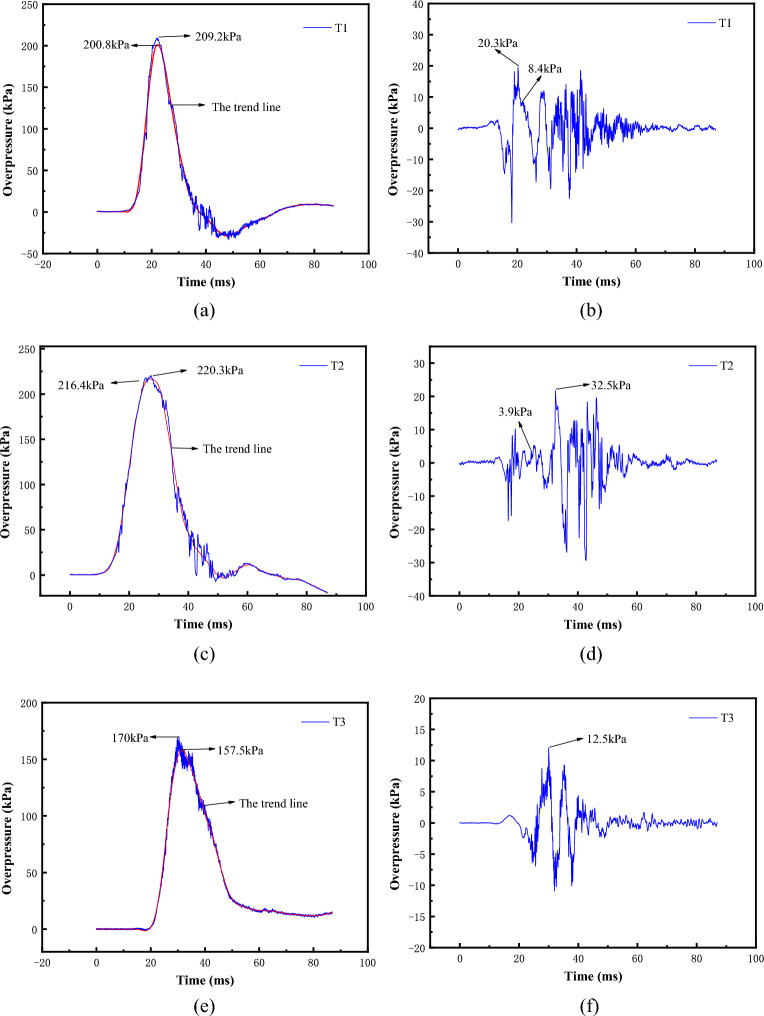
Overpressure, trend line and oscillation time history of each measuring point. (**a**) T1 overpressure and trend line time history, (**b**) T1 oscillation time history, (**c**) T2 overpressure and trend line time history, (**d**) T2 oscillation time history, (**e**) T3 overpressure and trend line time history, (**f**) T3 oscillation time history.

Figure 13 gives the overpressure time course curve of each measurement point in the test section. There are two stages of pressure rise and fall in the overpressure time course curve of the measurement points, and there are obvious pressure oscillations. After ignition at the bottom of the main pressure vessel, the combustible gas in the test section burns forward to generate pressure waves, which compresses the air in front of the wave front surface and leads to a gradual increase in the pressure at each measurement point. As the test section is open at one end only, the pressure release is relatively slow, coupled with the continuous heat exchange between the high-temperature and high-pressure gases in the vessel and the outside world, the shock wave pressure gradually decreases, with a duration of several tens of milliseconds.

The measured overpressure time-course curve in the semi-confinement case consists of two components, the trend line and the oscillation line, and can be considered as a combination of gas combustion explosion pressure rise and pressure oscillation. Due to the pipe wall constraints around the test section, the complex interactions between the shockwave and the pipe wall and between the shockwave and the shockwave resulted in oscillations in the overpressure time-course curve. At the T1 measurement point, the peak overpressure of the shock wave load rises from 200.8 to 209.2 kPa, with an increase of about 4.1%, and the maximum oscillation peak of 20.3 kPa occurs along the rising edge of the overpressure time course curve.At the T2 measurement point, the peak overpressure rises from 216.4 to 220.3 kPa, with an increase of about 1.8%, and the maximum oscillation peak of 32.5 The peak value of overpressure at measurement point T3 rises from 157.5 to 170 kPa, with an increase of about 7.9%, and the maximum oscillation peak value of 12.5 kPa occurs at the peak of the overpressure time curve. According to the trend line of each measurement point, the maximum rate of overpressurization at measurement points T1, T2, and T3 can be obtained, and the rate values are 18,267 kPa/s, 11,532 kPa/s, and 11,380 kPa/s, respectively.

#### Comparative analysis of results

**Figure 14:**
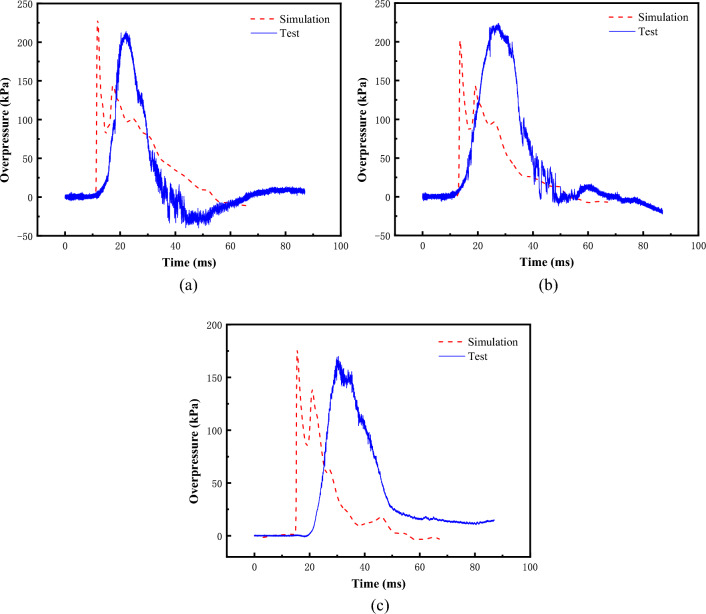
Comparison of results. (**a**) T1, (**b**) T2, (**c**) T3.

Figure 14 gives the gas explosion driving test and numerical simulation of the test section of the overpressure time course curve of each measurement point. The overpressure time course of the numerical simulation results agrees with the trend of the test results. In the semi-confined numerical simulation, the same rapid pressure rise occurs with the fuel burst. The neglect of heat exchange with the outside world in the numerical simulation results in a faster boosting rate in the numerical simulation. During the depressurization, there is no oscillatory depressurization in both the numerical simulation and the test. Table 4 shows the comparison between the test results and the numerical simulation results, from which it can be seen that the difference between the peak shock wave overpressure is all within 15%, the positive pressure duration has the largest difference of 46.8% at point T1, and the rest of the measurement points are all within 15%. In summary, it is believed that the numerical method as well as modeling can better simulate the gas explosion scenarios under different operating conditions and give credible gas explosion loads.

**Table 4:** Comparison between test results and numerical simulation results.

Test point		Test	Numerical simulation	Relative error
T1	Peak overpressure/kPa	200.8	228.7	13.8%
	Positive pressure duration/ms	25	47	46.8%
T2	Peak overpressure/kPa	216.4	202.3	6.5%
	Positive pressure duration/ms	37	41	9.7%
T3	Peak overpressure/kPa	157.5	175.2	11.2%
	Positive pressure duration/ms	35	40	12.5%

## Peak attenuation law of shock wave overpressure

**Figure 15:**
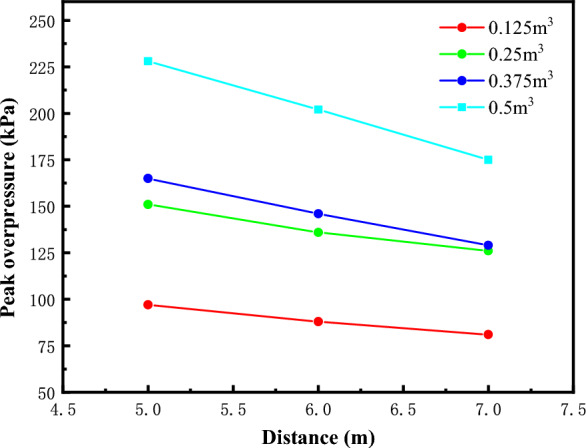
Variation curve of peak overpressure with distance.

In order to study the attenuation law of the shock wave overpressure peak in the test section, the change process of the shock wave overpressure peak with distance at each test point under different volumes is analyzed. First determine the main covariates affecting the explosion shock wave, and then use the method of magnitude analysis^26–30^ to derive the general function relationship between the peak shock wave overpressure and the propagation distance of the shock wave propagating along the axial direction of the test pipe. The peak overpressure decay process is shown in Fig. 15.

For the propagation of the shock wave in the test section pipe, if the viscosity and heat conduction of the medium are neglected, the main physical quantities affecting the peak overpressure of the air shock wave ∆*P* are: methane concentration *c* in the methane-oxygen mixture; gas cloud volume *V*; initial pressure of the air *p*_0_ = 100 kPa; L distance from the ignition source; and the cross sectional area of the test section *S*. ∆*P* is the quantitative quantity being measured, and *c*, *V*, *p*_0_, *L*, and *S* are the main quantitative quantities.

Then the peak overpressure of the explosive shock wave in the pipe can be expressed as:

12 $$ f(c,V,S,L,r,p_{0} ,\Delta P) = 0. $$

The *L*-*M*-*T* measure unit system was used to construct the measure matrix as shown in Table 5.

**Table 5:** Dimensional matrix.

Parameters	*λ*_1_	*λ*_2_	*λ*_3_	*λ*_4_	*λ*_5_	*λ*_6_
	*V*	*S*	*p*_*0*_	*L*	*c*	Δ*P*
*M*	0	0	1	0	0	1
*L*	3	2	− 1	1	0	− 1
*T*	− 20	0	− 2	0	0	− 2

From the table, among the 5 principal quantities, the maximum safe quantities with independent quantities are 2, and 2 principal quantities can be chosen among them: *p*_0_, *L*. According to *π*-theorem, the remaining quantities are *V*, *S*. The corresponding number of *π* is 2, and the corresponding *S* has:

13 $$ \pi_{1} = \frac{S}{{p_{0}^{{\alpha_{1} }} L^{{\alpha_{2} }} }}, $$

and

14 $$ \left[ {\pi_{1} } \right] = \frac{{L^{2} }}{{M^{{\alpha_{11} }} L^{{ - \alpha_{12} }} T^{{ - 2\alpha_{13} }} L^{{\alpha_{21} }} }} = 1, $$

can obtain *π*_1_ = *S*/* L*^2^.

Similarly corresponding to V there is *π*_2_ = *V*/*L*^3^ and for the quantized quantity ∆p there is *π* = ∆*P*/*p*_0_. From the above analysis two dimensionless *π* constants are obtained as in Eq. (15):

15 $$ \left\{ \begin{gathered} \pi_{1} { = }S/L^{2} \hfill \\ \pi_{2} { = }V/L^{3} \hfill \\ \end{gathered} \right.. $$

Then Eq. (15) can be transformed into *f*(*π*_1_*, π*_2_) = *π*, which leads to the basic attenuation of the peak explosion shock overpressure in the pipeline Eq. (16):

16 $$ \frac{\Delta P}{{p_{0} }} = f\left( {c,\frac{S}{{L^{2} }},\frac{V}{{L^{3} }}} \right). $$

The simplified model expression is:

17 $$ \frac{\Delta P}{{p_{0} }}{ = }c^{{\alpha_{1} }} \left( {\frac{S}{{L^{2} }}} \right)^{{\alpha_{2} }} \left( {\frac{V}{{L^{3} }}} \right)^{{\alpha_{3} }} . $$

Take the natural logarithm for both sides of the equation:

18 $$ \ln \frac{\Delta P}{{p_{0} }}{ = }\alpha_{1} \ln c + \alpha_{2} \ln \left( {\frac{S}{{L^{2} }}} \right) + \alpha_{3} \ln \left( {\frac{V}{{L^{3} }}} \right). $$

Equation (18) transforms the multivariate nonlinear fitting problem into a multivariate linear fitting problem, performs multivariate linear regression on the calculation results, calculates the unknown parameters *α*_1_, *α*_2_, *α*_3_ of which *p*_0_, *c*, *S*, *L*, *V* are the known quantities, and then obtains the equation of the peak overpressure attenuation of the shock wave in the pipeline of the test section after the collation:

19 $$ \frac{\Delta P}{{p_{0} }}{ = 2298}{\text{.926}}c^{7.050} \left( {\frac{S}{{L^{2} }}} \right)^{ - 0.505} \left( {\frac{V}{{L^{3} }}} \right)^{0.525} ,\,\,\left( {0.125m^{3} \le V \le 0.5m^{3} } \right),\,\,\left( {R^{2} = 0.95} \right). $$

## Conclusions

In this paper, a numerical model of methane blast driving is established by FLACS software, and the maximum driving load that can be achieved by methane blast in methane-air and methane-oxygen mixtures is investigated and verified by experiments, with the error within 15%. Finally, the empirical equations for the variation of the peak overpressure of the explosive air shock wave in the pipeline along the length of the pipeline under different volumes are given by the method of magnitude analysis. The conclusions are as follows:

1. In the methane-air mixture, the largest explosion pressure is observed at a methane body volume fraction of 9.5 vol%. As the volume ratio exceeds 9.5 vol%, there is a decrease in axial explosion pressure, while methane dosage and gas cloud length exhibit a non-linear increase in shockwave pressure. For a specific volume of gas cloud, the initial peak incident overpressure value is highest, followed by an accelerated attenuation rate of subsequent peaks. The maximum error in peak overpressure measurements at different points is found to be 34.6%, 32.3%, and 29.1%.
2. When the volume of methane increases, the oxygen content also increases. In comparison to air, the shock waves overpressure rises significantly faster; however, there is no noticeable change in positive pressure duration. The peak value of overpressure gradually increases, but its attenuation rate remains consistent with that observed in air.
3. In the methane-oxygen mixture, when the volume ratio of methane to oxygen is 1:2, the shock wave velocity exceeds the speed of sound, resulting in detonation. Furthermore, within a unit length, the attenuation coefficient maintains an approximate peak overpressure of 0.9 for the shock waves.

## Data Availability

The datasets used and analysed during the current study available from the corresponding author on reasonable request.
